# Gauging the Risk Factors for Asymptomatic Bacteriuria in Type-2 Diabetic Women: A Case-Control Study

**DOI:** 10.7759/cureus.9069

**Published:** 2020-07-08

**Authors:** Syed Muhammad Jawad Zaidi, Mehwish Kaneez, Talal Almas, Laiba Fatima, Hafiz Abu Safian, Ali Murad Jamal, Muhammad Zubair Satti, Rubaid A Dhillon, Abdullah Bin Zubair, Syed Faheem Bukhari

**Affiliations:** 1 Internal Medicine, Rawalpindi Medical University, Rawalpindi, PAK; 2 Internal Medicine, Royal College of Surgeons in Ireland, Dublin, IRL; 3 Cardiology, Rawalpindi Medical University, Rawalpindi, PAK; 4 Internal Medicine, Islamic International Medical College, Rawalpindi, PAK; 5 Internal Medicine, Rashid Latif Medical College, Lahore, PAK

**Keywords:** diabetes, urinary tract infection (uti), asymptomatic bacteriuria (asbu)

## Abstract

Background

There is conflicting literature pertaining to the risk factors of asymptomatic bacteriuria (ASBU) in diabetic women. ASBU is a well-established risk factor for frequent urinary tract infections (UTIs), and the risk factors that predispose diabetic women to ASBU should, therefore, be evaluated.

Objectives

This study aims to discern these aforesaid risk factors in type-2 diabetic women, define a population subset at particularly high risk for ASBU, and gauge the efficacy inherent in adhering to an antibiotic regimen in combatting ASBU.

Methods

An analytical, case-control study was conducted at the Diabetic Clinic of the Holy Family Hospital (HFH), Rawalpindi, Pakistan. The participants included were type-2 diabetic women reporting to the clinic for routine follow-up. Six hundred and sixty-seven urine samples from these type-2 diabetic women were evaluated. Positive cases were those in which patients were diagnosed with ASBU according to the guidelines, while those with no ASBU constituted the control group. Common risk factors for UTI were excluded in both groups. Age, socioeconomic status, hygiene practices, and contraceptive use were matched between cases and controls.

Results

Nineteen percent of type-2 diabetic women presented with ASBU in our study. The significant risk factors for ASBU were a higher HbA1c level (OR 1.97), more years since the initial diagnosis of diabetes (OR 1.49), a prior UTI history (OR 2.49), excessive antibiotic use (OR 2.72), sodium-glucose cotransporter-2 (SGLT2) inhibitor use (OR 1.75), and proteinuria (OR 1.88) in the multivariate model. Body mass index (BMI), age of the patients, pyuria, and voiding dysfunction manifested no association with ASBU. Antibiotic use was significantly associated with the type of bacterial species precipitating the ASBU.

Conclusion

The clinicians must keep in mind the association between the various patient parameters and ASBU, especially in prescribing antibiotics to diabetic women. More studies are needed to further elaborate on these risk factors and revise the patient management in at-risk cases for ASBU and UTIs.

## Introduction

Type-2 diabetes mellitus (T2DM) increases the risk of many infections. The urinary tract is the most common location for these infections that occur, in part, due to associated immune and nervous system defects caused by hyperglycaemia and partly by a glucose-rich environment (glycosuria) in the urinary tract [1-3]. This environment facilitates pathogenic growth and enhances bacterial resistance, predisposing diabetics to urinary tract infections (UTIs) [1]. UTIs add to the already exorbitant economic burden of T2DM and constitute significant morbidity and mortality among diabetics as they have recurrent, more severe, and refractory infections of the urinary tract. Additionally, more sinister complications, such as pyelonephritis, emphysematous UTIs, and sepsis, can also routinely ensue [2, 4-5]. UTIs are noted to be more common among diabetic females than males due to female gender-associated risk factors [6].

Another clinically related entity is asymptomatic bacteriuria (ASBU), which is defined as the isolation of the same bacterial species (greater than 10^5^ colony-forming units) on at least two occasions in aseptically collected, mid-stream urine samples from a subject with no symptoms of a UTI (dysuria, burning micturition, frequency, or urgency) [7]. ASBU incidence increases with age but is much greater in women than in men [8]. T2DM increases the risk of ASBU. An estimated 29% of T2DM women have ASBU, making it one of the most common complications of diabetes [9]. For reasons unknown, ASBU occurrence is similar in males with or without T2DM [8].

Whether every clinical occurrence of a UTI is preceded by ASBU is, of yet, unknown, but the presence of ASBU in diabetics is a proven risk factor for recurrent, symptomatic, and severe UTIs [10-12]. In women with T2DM, ASBU is recognized as the most important risk factor for clinical UTIs [13]. However, screening and treatment of ASBU in diabetics has remained at the epicentre of a medical conundrum. Many long-term studies have established that routinely treating ASBU in all patients adversely affects patients' health (due to the associated side-effects of antibiotics) and promotes bacterial resistance, a major global medical challenge [12, 14-15]. Thus, the current guidelines by the Infectious Diseases Society of America (IDSA) recommend against the screening and treatment of ASBU in diabetics [16].

Due to the clinical risk of ASBU in the form of UTI, the associated risk factors that herald the onset of ASBU are increasingly being studied. The IDSA guidelines also indicate the need for more research on ASBU-associated risk factors in greater detail, particularly in women [16]. The literature regarding risk factors of ASBU is abundant, but the results it delineates portray a contradictory picture. For example, it is clear that poor glycemic control increases the risk of UTIs, but its effect on the incidence of ASBU remains somewhat elusive. Interestingly, several studies report a significant association, while others indicate no correlation between glycemic control and ASBU [9, 11]. The other proposed risk factors also have elucidated contradictory results. A plausible explanation for these findings may be inappropriate matching, varying definitions of ASBU in studies, and heterogeneous study populations which make drawing meaningful comparisons an onerous task. 

This study considered the effect of various proposed risk factors, such as glycemic control (indicated by glycosylated hemoglobin (HbA1c) level), years since the initial diagnosis of diabetes, use of SGLT-2 inhibitors, use of penicillin, UTI history, voiding dysfunction, BMI, insulin use, proteinuria, and pyuria in the occurrence of ASBU in T2DM females after proper matching. We considered the definition of ASBU as recommended by IDSA. This study also observed the effect of antibiotic use on the bacterial species causing ASBU. The conflicting literature on various risk factors mandates studies like ours to further investigate the factors that predispose to the occurrence of ASBU. Such studies may provide new insights regarding the risk factors of ASBU, in enhancing its understanding, and identifying a diabetic population at high risk for ASBU and, hence, subsequent UTIs. Thus, the results may help in revising patient management of the selected population in accordance with our study. 

## Materials and methods

Study design

An analytical, case-control study was conducted from February 2019 to January 2020 in the Diabetic Clinic of the Holy Family Hospital (HFH), Rawalpindi, Pakistan. Urine routine examinations (RE) of T2DM females reporting to the clinic for follow-up of diabetes and not having any symptoms of a UTI were performed after obtaining written informed consent. The patients were advised regarding proper specimen collection and avoiding contamination. The specimens were then transported and analyzed according to the Clinical and Laboratory Standards Institute (CLSI) guidelines [17]. Urine culture was then performed in positive cases to isolate the bacterial strains. The details of other variables pertinent to the study, including age, diabetic years, and any therapies used for diabetes, were entered on the data collection curated for the research. Six hundred and sixty-seven samples were tested for six months with simultaneous documentation and interviews of patients regarded as cases. Ninety-three samples reported more than two bacterial species or more than 20 squamous cells and were rejected on the grounds of contamination. 

Selection of cases 

The procedure for diagnosing ASBU was as follows: In the first visit, 163 patients reported bacteriuria who were advised to report back the next day for another sample. On the second visit, 12 patients did not report and were excluded from the study. Twenty-four patients had no bacteriuria on the subsequent sample, while 127 patients reported bacteriuria in the second sample. These 127 patients were regarded as ASBU-positive. They were then interviewed in detail by trained interviewers to exclude pregnancy, sexual intercourse in the preceding week, insulin-dependent or type 1 diabetes mellitus (IDDM), catheterization of the urinary tract, structural urinary tract abnormalities, nephrolithiasis, chronic kidney disease (CKD), any surgical procedure in last four months, antibiotic use in last 14 days, hospital admission in the last four months, any organ failure, and conditions predisposing to immunosuppressive states (human immunodeficiency virus (HIV), malignancies, and steroid use). Such rigorous exclusion criteria were applied to exclude common causal factors of UTIs and allow proper matching. After scrutiny of the above factors, 83 cases were finally selected. 

Selection of controls 

The source of control was those diabetics who had negative findings on the first urine routine examination. Negative findings, in this case, referred to the absence of bacteriuria in diabetic patients and a concomitant unremarkable urine RE. The same exclusion criteria were applied to controls, and 166 controls (two controls per case) were then selected from the population after proper matching. 

Matching

Cases and controls were matched by age, socio-economic status, contraceptive use, and general hygiene practice (assessed by the educational level of the patient). Socio-economic matching was performed by dividing cases and controls into categories based on the monthly income of the family. Both cases and controls were then interviewed in detail by trained interviewers regarding the proposed risk factors, with the information entered on a standard data collection form developed for the study

Measurement of exposures

Diabetic years were measured as the number of years since the initial diagnosis of diabetes. Glycemic control was defined as good glycemic control (HbA1c less than 7%) and poor (HbA1c more than 7%) [11]. HbA1c levels were entered from the patients’ files if they were performed less than seven days before sample collection. Otherwise, this investigation was ordered at the time of sample collection to ensure that current HbA1c levels were correlated with ASBU. Insulin and SGLT2 inhibitor use were indicated as yes or no depending on whether the patient uses these for controlling diabetes. Antibiotic use was defined as “normal” when the patient used them only upon prescription and there were less than four prescriptions in the last six months. Contrarily, if the patient used antibiotics frequently, without prescription or had more than four prescriptions in the last six months, the scenario was labeled excessive. Voiding dysfunction was measured as yes or no based on whether a patient had complaints of retention, dripping, or incomplete emptying at the time of the interview. UTI history was measured as yes (an episode of UTI in last year) or no (no UTI episode in last year) [9]. Proteinuria and pyuria were also measured as yes or no depending upon the report of urine RE.

Statistical techniques

Eighty-three cases and 166 controls were selected for an alpha level of 0.05 and 80% power of the study. The descriptive analysis of study variables was performed by using Statistical Package for Social Sciences (SPSS), v23.0 (IBM SPSS Statistics, Armonk, NY, US). The normality of data was checked by the Shapiro-Wilk test. The Chi-squared test and independent samples t-test were used to find the association of risk factors for ASBU. A univariate analysis technique (separate binary logistic regression) was applied to check the odds of individual risk factors. To diagnose and exclude multicollinearity, a VIF (variance inflation factor) was calculated for all risk factors by entering two risk factors at a time in the regression model. Only the results with VIF values less than 5 were evaluated in the multivariate model, performed by using forward binary logistic regression. The odds ratio was calculated for a confidence interval (CI) of 95% and a p-value less than 0.05 was considered statistically significant.

## Results

Population parameters and characteristics of cases and controls are detailed in Table 1 below.

**Table 1:** An Illustration of the Population Parameters and the Matching Characteristics

Variable	Characteristics of cases	Characteristics of controls
Total samples tested: 667	n = 83	n = 166
Mean age	59.3 ± 3.1	57.7 ± 2.9
Mean family income	Rs 37,581 ± 2,198	Rs 36,987 ± 1,783
Educational status	Under matric: 40 (48.2%); above matric: 43 (51.8%)	Under matric: 79 (47.6%); above matric: 87 (52.4%)
Contraceptive use cases	8 (9.6%)	19 (11.4%)

Table 2 further delineates the difference in cases and controls with pertinence to the risk factors. To test the association of risk factors with ASBU, a chi-squared test was performed for categorical risk factors, and an independent samples t-test was performed for quantitative risk factors.

**Table 2:** An Elucidation of the Association of the Varying Risk Factors With ASBU *Chi-square test **Independent samples t-test ASBU: asymptomatic bacteriuria; BMI: body mass index; HbA1c: glycosylated hemoglobin; SGLT2: sodium-glucose cotransporter-2; UTI: urinary tract infection

Parameter		Cases	Controls	P-value*
BMI	< 18.5 Kg/m^2^	15 (18.0%)	31 (18.67%)	0.926
	18.5 to 24.9 Kg/m^2^	42 (50.6%)	87 (52.4%)	
	25 to 30 Kg/m^2^	26 (31.3%)	48 (28.9%)	
Insulin use		37 (44.6%)	79 (47.6%)	0.23
SGLT2 use		41 (49.4%)	57 (34.3%)	0.02
Antibiotic use	Normal	42 (50.6%)	118 (71.1%)	0.001
	Excessive	41 (49.4%)	48 (28.9%)	
Voiding dysfunction		39 (47.0%)	91 (52.8%)	0.244
UTI history		31 (37.3%)	27 (16.3%)	< 0.001
Proteinuria		32 (38.6%)	25 (15.1%)	< 0.001
Pyuria		14 (16.9%)	19 (11.4%)	0.234
For quantitative variables				
Parameter		Cases	Controls	P-value**
HbA1c level (mean)		9.91 ± 1.7	6.14 ± 0.92	0.002
Diabetic years (mean)		11.8 ± 2.1	8.9 ± 1.3	< 0.001
Age (mean)		59.3 ± 3.1	57.7 ± 2.9	0.43

Furthermore, Table 3 delineates the results of univariate analysis of risk factors for ASBU. 

**Table 3:** Results From the Univariate Analysis Performed for all the Proposed Risk Factors BMI: body mass index; CI: confidence interval; HbA1c: glycosylated hemoglobin; SGLT2: sodium-glucose cotransporter-2; UTI: urinary tract infection

Variable	Odds Ratio (Exp B)	95% CI	p-value
BMI	1.33	0.45 - 2.92	0.73
Age	1.11	0.20 - 2.82	0.17
Insulin use	1.12	0.15 - 2.97	0.16
SGLT2 use	1.26	0.58 - 3.34	0.01
Antibiotic use	1.71	0.24 - 3.98	0.02
Voiding dysfunction	1.04	0.02 - 2.34	0.34
UTI history	1.62	0.65 - 3.89	< 0.001
Proteinuria	1.54	0.30 - 3.67	< 0.001
Pyuria	1.12	0.19 - 2.89	0.51
HbA1c level	1.43	0.17 - 3.21	0.007
Diabetic years	1.37	0.21 - 3.43	0.004

In Table 4, the factors significant for ASBU in multivariate analysis (performed by multiple binary logistic regression) are shown, along with the odds ratio. We found that HbA1c had VIF values greater than 5 for insulin use, BMI, and age of the patients. As a result, age and insulin use were excluded from the multivariate model, as delineated in Table 4. 

**Table 4:** Significant Risk Factors as Determined by the Multivariate Analysis (Binomial Logistic Regression) CI: confidence interval; HbA1c: glycosylated hemoglobin; SGLT2: sodium-glucose cotransporter-2; UTI: urinary tract infection

Variable	Odds Ratio (Exp B)	95% CI	p-value
SGLT2 use	1.75	0.13-3.98	0.03
Antibiotic use	2.72	1.02-4.67	0.01
UTI history	2.49	0.97-4.45	<0.001
Proteinuria	1.88	0.75-3.88	<0.001
HbA1c level	1.97	0.93-3.92	< 0.001
Diabetic years	1.49	0.32-3.10	0.001

Lastly, Table 5 delineates the type of bacterial strains significantly associated with antibiotic use. Excessive antibiotic use is associated with ASBU and results in an increased incidence of *Klebsiella* or *Pseudomonas* species infection. 

**Table 5:** Cross-Tabulation of Bacterial Strain With Antibiotic Use The p-value of chi-square test was 0.002

Antibiotic use in cases	Type of organism				
	Escherichia coli	Klebsiella	Pseudomonas	Staphylococcus saprophyticus	Staphylococcus aureus
Normal = 42 (50.6%)	32 (76.2%)	2 (4.8%)	1 (2.4%)	4 (9.5%)	3 (7.1%)
Excessive = 41 (49.4%)	19 (43.9%)	10 (24.4%)	9 (19.5%)	2 (4.9%)	1 (2.4%)
Total cases	51 (61.4%)	12 (14.4%)	10 (10.8%)	6 (7.2%)	4 (4.8%)

## Discussion

Our study provides important results regarding the risk factors of ASBU. The prevalence of ASBU was approximately 19% (127 out of 667 samples tested were positive) which is similar to values obtained from other studies [9, 18]. Since we performed a rigorous matching of cases and controls with an ample sample size, there is a less likelihood of confounding the effect of risk factors on the overall results. 

The first factor evaluated was glycemic control as indicated by HbA1c levels. HbA1c levels are considered a reliable marker of blood glucose concentration. A high HbA1c indicates poor glycemic control in diabetes. Its association with UTI is proved by many studies and the plausible explanation is the associated glycosuria that accompanies higher blood glucose levels and facilitates pathogen growth and resistance [19]. However, its effects on the occurrence of ASBU are less clear. Logically, the same explanation should hold here, that is, a higher HbA1c should translate into a higher risk for ASBU. In our study, there was a significant association of these factors (p = value of the chi-squared test was < 0.001), and in multivariate analysis, HbA1c had the odds ratio of 1.97 (p < 0.001). In other words, each unit increase in HbA1c leads to about two times an increased likelihood of ASBU, as elucidated in our study. However, the literature is replete with reports yielding conflicting results regarding this association. The results of some studies support our findings of a positive association, whereas a few studies report no association between HbA1c levels and ASBU occurrence [8, 11, 20-21].

We found no association of age with ASBU occurrence among the cases. Thus, advanced age itself does not lead to an increased risk of ASBU. The study by Bonadio et al. [20] also reported the same result regarding age, but Geerlings et al. showed in their study that age is a risk factor for ASBU [9].

Diabetic years are the number of years since diagnosed with diabetes, and our study indicated that a longer duration of diabetes led to a higher risk of ASBU among the subjects. In multivariate analysis, each unit increase in diabetic years led to about 1.5 times more chances of ASBU. These results contrast with the study by Boroumand et al., who reported no association of diabetic years with ASBU in Iranian T2DM women [18].

In our study, we observed no significant difference in BMI between cases and controls. The results of the study conducted by Ishay et al. [21] support our finding, whereas Geerlings et al. identified low BMI as a risk factor for ASBU [9]. Insulin use also showed no significant association with ASBU, and the study by Bonadio et al. supports our results [20]. We also found that voiding dysfunction does not confer an increased risk for ASBU, which further corroborates the finding reported by Geerlings et al. [9].

A prior history of UTI has been associated with an increased risk of ASBU by Geerlings et al., and our results are in agreement with these findings [9]. In multivariate analysis, we found that past UTI history increases the chances of ASBU by about 2.5 times (p < 0.001).

Next, we evaluated pyuria as a risk factor for ASBU. Pyuria is considered significant at more than 10 leukocytes per mm of urine [22]. Our study indicates no association between ASBU and pyuria. Only 14 cases had pyuria, and interestingly, 19 controls also reported pyuria [22]. The finding that pyuria is not significantly associated with ASBU implies the absence of inflammation in ASBU, which contrasts with symptomatic UTIs. 

Proteinuria in diabetes is prevalent due to continuously declining kidney function, which is yet symptomless. Our cases had a significant association with proteinuria but proteinuria in this setting might just be an associated complication of diabetes and not a risk factor for ASBU. Similar results are reported by a systemic review that does associate ASBU with proteinuria but argues that a causal relation between ASBU and proteinuria cannot be proved after analyzing many studies [23]. However, further research is needed to identify the role of proteinuria in the context of UTI in diabetics.

The patients on SGLT2 inhibitors had a significantly higher risk of developing ASBU in our study. The association between SGLT2 inhibitors and UTI has been established, and the reason is the mechanism of action of these drugs, to inhibit renal glucose uptake and lead to pharmacologically-induced glycosuria which confers increased risk for UTI [3]. However, we found no study that correlated the use of SGLT2 inhibitors with ASBU. The finding indicates that SGLT2 inhibitors need to be investigated in more detail regarding their interaction with the host microbiome.

Antibiotic use among diabetics increases the risk of ASBU development. The chi-squared test for the association was significant (p-value: 0.001). Further, when the culture results were analyzed, we found that antibiotics also affected the bacterial species causing ASBU (p-value: 0.002). Cases with excessive antibiotic consumption had a higher incidence of *Klebsiella* and *Pseudomonas* strains in the urine. The most common organism associated with ASBU is *E. coli *[24]. *Klebsiella* and *Pseudomonas* are rather uncommon causative agents of ASBU in the population. However, in cases with excessive antibiotic use, we found that their occurrence was much more significant, accounting for about 24% and 20% of all ASBU cases, respectively. The logical explanation is the fact that many antibiotics are excreted by kidneys and have the potential to affect the normal commensals by altering the host-microbiota relation [25]. When used excessively, antibiotics lead to the selection of resistant pathogens and colonization by relatively rare and more resistant bacteria, such as the *Klebsiella* or *Pseudomonas* species identified in our study. The finding calls for a strict antibiotic stewardship practice when treating infections in the diabetics. Diabetics are prone to frequent infections, particularly of the respiratory tract such as tonsillitis and bronchitis, which are relatively difficult to treat in diabetics due to associated immune defects caused by hyperglycemia [19]. Hence, the clinicians tend to prescribe broad-spectrum antibiotics, particularly in developing countries, where there is no strict adherence to antibiotic stewardship at the national level [14]. Because many ASBU cases eventually translate to UTIs, diabetics who are prescribed frequent broad-spectrum drugs are at increased risk of UTIs by more resistant organisms. These resistant pathogens can become problematic to treat and potentially increase healthcare costs, which can put additional strain on the already overburdened health system of developing countries. Thus, keeping in mind this potential hazard of antibiotic abuse, new guidelines need to be developed specifically regarding antibiotic prescription to diabetics.

All of the above findings become clinically more important if one bears in mind the fact that ASBU does translate to symptomatic and severe UTIs in diabetic females and these UTIs impair the patient’s quality of life [9, 13, 26]. Thus, the researchers stress to think of ASBU more as a risk factor rather than an incidental finding, as some clinicians tend to argue. The clinicians should aim at identifying the high-risk ASBU patients and assess whether targeting this population subset pharmacologically benefits them or not. Our study is an attempt to facilitate the first part of the same objective, for we need to be more certain regarding the diabetics who are prone to ASBU. Moreover, the next step will be to identify the factors that underlie the conversion of ASBU to UTIs, since not all diabetics with ASBU develop UTIs. A deeper understanding of such yet arcane factors requires more studies like ours to understand the mechanism and risk factors for ASBU. 

The co-occurrence of these different risk factors in the same patient will undoubtedly have important clinical implications. Diabetic women with poor glycemic control and longer diabetic duration, having a history of UTIs, along with proteinuria and excessive antibiotic use, will be at higher risk for ASBU and subsequent UTIs. These risk factors must be considered while dealing with such patients so that comprehensive patient management is ensured. As an example, the clinicians should avoid prescribing SGLT2 inhibitors to this patient population because it will further increase the risk of developing ASBU. We understate the need to develop a predictive model based on these risk factors and then assess its predictive efficiency in different population subsets. 

The above discussion highlights the need for more extensive studies and cohorts to evaluate the proposed risk factors in detail and see whether different population characteristics affect the occurrence of ASBU. Although routine screening of ASBU is contraindicated due to lack of benefits to the patients, once the risk factors of ASBU are established by more studies like ours, it may help to identify a diabetic population at risk for ASBU and UTIs. Screening and treatment in such cases can boast a prophylactic value, eventually leading to the provision of ameliorated medical care to diabetic patients.

## Conclusions

The factors that herald the onset of ASBU were concluded to be higher HbA1c levels, more diabetic years, a UTI history, excessive antibiotic use, the use of SGLT2 inhibitors, and proteinuria. Contrarily, BMI, age of the patient, insulin use, pyuria, and voiding dysfunction showed no association with ASBU. Excessive antibiotic use also influenced the presence of certain bacterial species, leading to an increased incidence of *Klebsiella* and *Pseudomonas* strains in the cultures obtained from ASBU patients. More studies are needed to define the factors that lead to ASBU and revise patient management in at-risk cases of ASBU and UTIs. 
